# Neotropical Sylvatic Mosquitoes and *Aedes aegypti* Are Not Competent to Transmit 17DD Attenuated Yellow Fever Virus from Vaccinated Viremic New World Non-Human Primates

**DOI:** 10.3390/v14102231

**Published:** 2022-10-11

**Authors:** Rafaella Moraes de Miranda, Rosilainy Surubi Fernandes, André Tavares da Silva-Fernandes, Anielly Ferreira-de-Brito, Silvia Bahadian Moreira, Renata Carvalho Pereira, Ygara da Silva Mendes, Sheila Maria Barbosa de Lima, Alcides Pissinatti, Marcos da Silva Freire, Jerônimo Augusto Fonseca Alencar, Ricardo Lourenco-de-Oliveira

**Affiliations:** 1 Instituto Oswaldo Cruz, Fundação Oswaldo Cruz, Rio de Janeiro 21040-900, Brazil; 2 Instituto de Tecnologia em Imunobiológicos/Bio-Manguinhos, Fundação Oswaldo Cruz, Rio de Janeiro 21040-900, Brazil; 3 Centro de Primatologia do Rio de Janeiro—CPRJ, Instituto Estadual do Ambiente, Guapimirim 25940-000, Brazil

**Keywords:** non-human primates, YF 17DD, vector competence

## Abstract

Beside humans, thousands of non-human primates (NHPs) died during the recent outbreak caused by the yellow fever virus (YFV) in Brazil. Vaccination of NHPs against YFV with the YF 17DD attenuated virus has emerged as a public health strategy, as it would reduce sylvatic transmission while also preserving endangered susceptible species. The hypothesis of establishing an uncontrolled transmission of this attenuated virus in nature was raised. We assessed vector competence of four sylvatic mosquito species, *Haemagogus leucocelaenus*, *Haemagogus janthinomys/capricornii*, *Sabethes albiprivus*, and *Sabethes identicus*, as well as the urban vector *Aedes aegypti* for YF 17DD attenuated vaccine virus when fed directly on eleven viremic lion tamarins or artificially challenged with the same virus. No infection was detected in 689 mosquitoes engorged on viremic lion tamarins whose viremia ranged from 1.05 × 10^3^ to 6.61 × 10^3^ FFU/mL, nor in those artificially taking ≤ 1 × 10^3^ PFU/mL. Low viremia presented by YF 17DD-vaccinated New World NHPs combined with the low capacity and null dissemination ability in sylvatic and domestic mosquitoes of this attenuated virus suggest no risk of its transmission in nature. Thus, vaccination of captive and free-living NHPs against YFV is a safe public health strategy.

## 1. Introduction

Yellow fever is an acute, hemorrhagic infectious disease transmitted through the bite of infected mosquitoes that occur in Africa and South America. It is caused by the yellow fever virus (YFV), genus *Flavivirus*. Transmission takes place in two main cycles, urban and sylvatic, with the latter only recently recorded in South America [1], where arboreal mosquitoes of genera *Haemagogus* and *Sabethes* are the main vectors [2].

According to the World Health Organization the world is currently experiencing a period of increased death risks due to YFV. In 2015–2016, Angola reported the resurgence of urban YFV in a serious epidemic that reached the Democratic Republic of Congo and imported cases reported in distant countries with low vaccination coverage, such as China [3,4]. Almost at the same time, a re-emergence of sylvatic YFV was detected in the Brazilian extra-Amazon region, resulting in the most severe epidemic in the country’s recent history. With this, in 2017–2019, a YFV wave affected areas with no record of virus circulation for more than seven decades and very low vaccination coverage, such as metropolitan areas in the Brazilian Southeast. About 14,000 suspected human cases were reported, 2259 (6.2%) laboratory-confirmed, with 773 deaths, comprising a lethality rate of 34.2% [5]. The mosquitoes *Haemagogus janthinomys*/*capricornii* and *Haemagogus leucocelaenus* were the primary vectors in this outbreak, while *Sabethes chloropterus* and some opportunistic *Aedes* species played local or secondary transmission roles [6,7].

YFV was isolated for the first time in 1927 from a blood sample of a young African man named Asibi. After 176 passages of the wild-type Asibi YFV strain in mouse and chicken embryo tissues, viscerotropism, and the ability of the virus to be transmitted by the domestic vector *Ae. aegypti* were lost [8]. On the other hand, the obtained attenuated strain, named 17D, maintained the ability of inducing immune response and protection in both humans and non-human primates (NHPs). Several substrains derived from the 17D strain were tested to reach an adequate attenuation level while maintaining immunogenicity [9], and the 17D-204 and 17DD substrains, which are respectively at passages 235–240 and 287–289, are currently employed worldwide [10,11,12]. The attenuated YFV vaccine currently used in Brazil is YF 17DD, which shares 99.9% of its nucleic acid sequence with 17D-204 [12,13,14].

The few evaluations of vector competence of *Ae. aegypti* for 17D attenuated vaccine virus concluded that it is able to infect the midgut epithelial cells of this mosquito species. However, unlike wild YFV strains, it has lost the ability to disseminate to secondary mosquito tissues and be transmitted through mosquito bites [15,16]. Thus, after ingestion by the mosquito, infection by the attenuated 17D strain, if established, would be limited to the *Ae. aegypti* midgut. To the best of our knowledge, no assessment of vector competence of wild Neotropical mosquito species for the attenuated virus 17D or 17DD nor of *Ae. aegypti* for the 17DD strain is available.

Thousands of epizootics in NHPs were recorded during the 2017–2018 YFV outbreak in Southeast Brazil. A total of 1177 NHP deaths were reported in the state of Rio de Janeiro (RJ) only. Howler monkeys (*Alouatta guariba clamitans*) and some endangered endemic NHP species, such as golden lion tamarins (*Leontopithecus rosalia*) were severely affected [17,18,19]. These events had significant repercussions and led to major concerns for NHP conservation programs [20]. Thus, evaluations of immunogenicity and safety of 17DD attenuated yellow fever vaccine currently applied to humans in Brazil in NHPs were promptly initiated, aiming at preventing the death of endangered species and reducing epizootics [21,22].

New World NHPs injected with wild-type YFV strains may display elevated viremia, usually within the first 3–5 days post-infection [17]. Some dead NHPs recovered during the 2017–2018 epizootics in Southern Brazil presented high viral loads in tissues like liver and blood [18,19]. If New World NHPs are similarly sensitive to the 17DD attenuated vaccine virus, they could infect mosquitoes, as free-living and even captive vaccinated animals can be bitten daily by dozens of mosquitoes.

In this context, this study evaluated the ability of YF 17DD attenuated vaccine virus to infect and be transmitted by mosquitoes fed vaccinated New World NHPs during viremia and artificially and orally challenged with the same viral strain at different viral loads.

## 2. Material and Methods

### 2.1. NHPs Used in the Study

Mosquitoes were fed on eleven vaccinated adult male and female lion tamarins (Family Callitrichidae, genus *Leontopithecus*) belonging to three species (3 *L. rosalia,* 6 *L.* *chrysomelas* and 2 *L.* *chrysopygus*). The animals were maintained in large outdoor enclosures at the Rio de Janeiro Primatology Center (CPRJ), located in Guapimirim, RJ, whose main aim is to breed endangered New World NHPs, such as lion tamarins and muriquis, for research and conservation efforts. The eleven flavivirus-naïve lion tamarins were inoculated with a single dose of 1 × 10^3^ plaque-forming units (PFU) (animal numbers 2266, 2435, 3110, 3503, 3504, 3533, 3617 and 3654) or 5 × 10^3^ PFU (animal numbers 3408, 3595, and 3644) of YF 17DD live attenuated vaccine produced by Bio-Manguinhos/Fiocruz (vaccine batch number 186VFA057Z), as detailed previously [22]. On the 3rd day post-vaccination, the animals were anesthetized with an intramuscular combination of ketamine (10 mg/kg) and midazolam (1 mg/kg) [23]. During anesthesia, a 1 mL blood sample was obtained to determine viremia and the mosquitoes were allowed to feed directly on the animals. When recovered from anesthesia, the lion tamarins were carefully returned to their respective enclosures.

The choice of the 3rd day for the mosquito oral challenge was based on previous studies with the wild virus (Asibi) and attenuated vaccines in monkeys usually employed in YFV vaccines assays (green monkeys and rhesus) as well as howler monkeys, with the viremia peak generally recorded from days 2 to 4 post-inoculation, depending on the injected dose and inoculation route [22,24,25,26].

### 2.2. Mosquitoes

Five mosquito species were used, namely the sylvatic species: *Hg. leucocelaenus*, *Hg. janthinomys/capricornii*, *Sabethes albiprivus*, and *Sabethes identicus*, and the domestic *Ae. aegypti.* Although *Ae. aegypti* is not expected to bite NHPs in the forest, it was included because previous vector competence evaluations for the 17D attenuated vaccine virus have been conducted on this species [15,16,27,28,29]. The populations of all challenged mosquito species originated from RJ. The *Sa. albiprivus*, *Sa. identicus*, and *Ae. aegypti* mosquitoes were obtained from colonies maintained in an insectary under controlled temperature, humidity, and photoperiod conditions (26 ± 1 °C, 70 ± 10% RH, 12h light-dark cycle), as detailed previously [20,30,31]. As they do not colonize under laboratory conditions, the adult *Hg. janthinomys/capricornii* and *Hg. leucocelaenus* employed herein were obtained from laboratory rearings of field collected eggs from a YFV-free area using ovitraps and treated as previously described [32]. Adult mosquitoes were maintained with access to 10% sucrose solution *ad libitum* for 24 h prior to ingesting the infected blood meal. Then, 60 five to seven days post-emergence female mosquitoes of each species were placed in cylindrical plastic boxes screened on top for the oral challenge with the virus, either directly on primates or artificially.

### 2.3. Blood Meal on Vaccinated NHPs

Depending on mosquito availability, each vaccinated lion tamarin was simultaneously exposed for 15 min to two to four mosquito cages, each containing 60 females of one of the aforementioned species. To this end, the cages were placed directly in contact with the epidermis of the lion tamarins, either on the lower abdomen or the inner thigh, which are naturally less hairy areas.

### 2.4. Artificial Laboratory Mosquito Oral Challenge

*Hg. leucocelaenus* and *Ae. aegypti* females treated as described above were fed a mixture of rabbit erythrocytes and YF17DD virus culture supernatant in the laboratory at three final dilutions, as previously described [31]. The applied viral stock was obtained after one passage of the YF 17DD live attenuated vaccine from the same batch used for lion tamarin vaccination onto Vero cells maintained with Earle’s 199 medium supplemented with 5% fetal bovine serum (FBS) under a 5% CO_2_ atmosphere and incubated for three days at 37 °C. A viral titer of the stock was evaluated by focus-forming unit (FFU) assays of serial dilutions on Vero cells and expressed as FFU/mL. The viral titer of the obtained stock was of 8.13 × 10^7^ FFU/mL. The stock was diluted in one part of Leibovitz L15 medium supplemented with 2% FBS and two parts of washed rabbit erythrocytes at final titers of 1 × 10^2^, 1 × 10^3^ and 1 × 10^4^ FFU/mL just before being offered to mosquitoes through a pig-gut membrane covering the base of feeders containing the infectious blood-meal maintained at 37 °C. Mosquito feeding was limited to 40 min.

### 2.5. Mosquito Screening and Incubation

After blood feedings directly on vaccinated lion tamarins or artificially, mosquitoes were immediately screened, and only fully engorged specimens were incubated under controlled temperature, humidity, and photoperiod conditions (26 °C; 80% RH; 12 h light-dark cycle) with daily access to 10% sucrose or honey solutions. When available, mosquitoes of each species were examined for vector competence 14 or 21 days after virus exposure (hereafter abbreviated as d.p.i.) following feeding on lion tamarins and 14 d.p.i. when artificially fed, as described previously [31].

### 2.6. Mosquito Examination

For infection and dissemination rate determinations, the body (abdomen + thorax) and head of each mosquito were respectively ground in 300 and 250 mL of Leibovitz L15 medium supplemented with 4% FBS and centrifuged at 10,000× *g* for 10 min at 4 °C. A 140 µL aliquot of the supernatant was used for RNA extraction employing the QIAamp Viral RNA Mini Kit following the manufacturer’s protocol. The extracted RNA from each mosquito tissue was tested in duplicate by a RT-qPCR capable of detecting both wild and attenuated vaccine virus genomes using previously described primers and protocols [6,33].

Saliva was collected to be analyzed to assess transmission only if infection and dissemination were confirmed as described previously [31].

The infection rate (IR) corresponds to the percentage of infected mosquitoes among the engorged ones, while the dissemination rate (DR) comprises the percentage of positive heads among mosquitoes with positive bodies, and the transmission rate (TR) corresponds to the proportion of individuals with positive saliva among those in which the virus disseminated to the head [34].

## 3. Results

### 3.1. Mosquitoes Were Not Infected when Fed Viremic YF 17DD-Vaccinated Lion Tamarins

The viral titers in the blood of vaccinated lion tamarins at the moment of the mosquito blood-feedings ranged from 1.91 × 10^2^ to 6.61 × 10^3^ FFU/mL (Table 1). None of the 689 mosquitoes belonging to the five species (319 *Ae. aegypti*, 141 *Hg. leucocelaenus*, 147 *Sa. albiprivus*, 77 *Sa. identicus*, and 5 *Hg. janthinomys/capricornii*) engorged on viremic lion tamarins became infected with 17DD attenuated vaccine virus, regardless of incubation time and injected vaccine dose. Most mosquitoes (82.3%) of the species that fed on all primates were examined at 14 d.p.i., and 122 *Ae. aegypti* that fed on four out of eight viremic animals were examined at 21 d.p.i.

Although infection is a prerequisite for virus dissemination to secondary mosquito tissues, we also analyzed the heads of mosquitoes of three species that fed on the two lion tamarins (# 2435 and 3617) with the highest blood viral titers. All, however, tested negative (Table 1).

### 3.2. Infection and Dissemination Rate Determinations of Laboratory Orally Challenged Mosquitoes

In total, 156 mosquitoes artificially fed blood infected with YF 17DD attenuated vaccine virus at three viral titers were tested (Table 2). No infection or dissemination was detected in any of the mosquitoes fed the mixture containing viral titers of 1 × 10^2^ and 1 × 10^3^ FFU/mL. Only one of the 96 mosquitoes of two species orally challenged with the titer of 1 × 10^4^ FFU/mL became infected, one *Hg. leucoceleanus* (IR = 2.7%), and no dissemination was detected.

Since infection and dissemination are prerequisites for salivary gland infection, we did not examine the saliva of mosquitoes fed on NHPs or artificially challenged.

## 4. Discussion

To the best of our knowledge, previous assessments of vector competence for YF attenuated vaccine virus have been performed only with the 17D strain and on only one mosquito species, the domestic *Ae*. *aegypti*. Thus, the present study is the first to employ the YF 17DD substrain to challenge wild mosquitoes, such as *Sa. albiprivus*, *Sa. identicus*, *Hg. janthinomys/capricornii*, and *Hg. leucocelaenus*, besides comparatively testing the urban vector, *Ae. aegypti*. Assessing vector competence of these mosquitoes in transmitting the attenuated virus from vaccinated viremic monkeys is essential and a prerequisite for risk assessments in the context of the wild animal vaccination initiatives.

The vaccination of NHPs against YFV has emerged as a public health strategy, aiming to aid in reducing transmission and slowing the spreading of YFV in forest fragments, in addition to preserving endangered species [35,36]. The safety and immunogenicity of the 17DD attenuated vaccine virus in New World NHPs began being tested in late 2018 in Brazil [22]. However, significant concerns were raised, as most New World NHP species are very sensitive to YFV and may develop high viremia loads when infected with wild-type virus strains. In nature, viremic vaccinated NHPs would be certainly bitten by arboreal mosquitoes competent to transmit the wild-type YFV. However, the vector competence of New World YFV vectors for the attenuated vaccine viruses 17D or 17DD had never been assessed. The hypothesis of establishing an uncontrolled transmission of the attenuated vaccine virus in the wild, affecting the safety characteristics and genetic stability of the vaccine, made this a significant concern. This scenario of sylvatic vaccine virus transmission would increase the risk of recombination between wild-type and vaccine viruses in potential co-infections or super-infections of wild and attenuated vaccine viruses in sylvatic mosquitoes during YFV epizootics. Flaviviruses can recombine, and recombination between attenuated vaccine viruses and a wild virus could result in a virus with potentially undesirable properties, such as reversion to the virulent phenotype [37].

Our findings, however, indicate that the probability of these phenomena occurring seems essentially null. Accordingly, *Ae. aegypti* and, most importantly, the *Haemagogus* and *Sabethes* species, respectively the primary and secondary sylvatic YFV vectors in South America, are not competent to transmit YF 17DD attenuated vaccine virus when feeding directly on viremic lion tamarins or artificially fed a blood meal containing the same virus substrain. Crucially, none of the five mosquito species even became infected when following feeding on viremic YF 17DD-vaccinated lion tamarins. The same result was obtained for mosquitoes artificially fed blood meals containing the YF 17DD attenuated virus from the same batch used to vaccine the primates at similar titers. Infection only took place, albeit at very low rate, when the artificial blood meal contained 1 × 10^4^ FFU/mL, a viral titer value not detected in the blood of any of the YF 17DD vaccinated New World NHPs, whether tamarins or howler monkeys, whose highest viremia titers were 6.61 × 10^3^ FFU/mL and 7.58 × 10^2^ FFU/mL (2.88 log_10_ FFU/mL), respectively [22]. Low viremia in 17DD-vaccinated primates have been previously reported in rhesus specimens injected with the same attenuated virus [24,38].

Arbovirus transmission by mosquitoes depends on the genetic characteristics of both the vector population and the virus [39,40], as well as on non-genetic factors, such as incubation temperature and the viral loads in the blood of the infected meal [41,42]. As the tested mosquito species and populations are known to be competent to transmit wild-type YFV of different genotypes and lineages when incubated at the same temperature employed herein [30], two main hypotheses was raised to explain the null to low IRs observed and, most importantly, null dissemination, a prerequisite for virus transmission: (a) low virus titers in the blood of vaccinated NHPs, and (b) the genetic properties of the attenuated virus.

It is known that the viral titers in infective meals influence both mosquito infection rates and the extrinsic incubation period [16,29,43]. Regardless of the vaccinated NHP species, viremia in lion tamarins and howler monkeys did not even reach 1 × 10^4^ FFU/mL.

Whitman (1939) [15] demonstrated that female *Ae. aegypti* that fed on YF-17D vaccinated volunteers and rhesus monkeys did not become infected with the vaccine virus. The author reported that human volunteers and part of the vaccinated rhesus monkeys that served as the source of infection for the mosquitoes exhibited low viremia. Even *Ae. aegypti* that fed on rhesus with viremias higher than those observed in 29 humans did not become infected. In view of this result, the author [15] suggested that the amount of virus circulating in vaccinated humans and rhesus was too low to infect *Ae. aegypti*.

Thus, the low titers in the blood of vaccinated New World NHPs tested herein and in rhesus previously injected with YF 17DD or 17D attenuated viruses [22,24,38] may explain failure in virus dissemination and transmission compared to the wild-type virus. Furthermore, besides low viremia, the genetic properties of the attenuated virus also play an important role in vector competence.

Although the YFV cycle in the mosquito is well known, the role of viral genetics in this process has not yet been fully elucidated. Vector competence for arboviruses depends mainly on vector-virus interactions governed by genotype-by-genotype interplays [34,39,40,43,44]. The attenuated vaccines 17 D (17D-204) and 17DD share 99.9% of their nucleotides, with 17D differing by 68 out of 10,862 nucleotides (approximately 0.63%) from the mosquito-transmitted wild-type Asibi strain from which it originated, resulting in 32 different amino acids [8,12,13,14]. The highest number of mutations [N = 42] is noted in the gene that encodes the E protein, and it is believed that mutations in this protein may be responsible for viral tropism alterations. The genomes of the two vaccine substrains (17D-204 and 17DD) do not differ from the wild-type Asibi virus in the 5′-terminal region or in the capsid, but instead in the 3′-UTR [45]. Studies have mostly focused on the E protein gene, specifically the putative E cellular receptor III binding domain, which contains important Flavivirus viral dissemination and transmission determinants, including YFV by *Ae. aegypti*. This indicates that certain genetic determinants, especially YFV dissemination in *Ae. aegypti*, must be located in non-structural protein genes or in the 3′ non-coding region (NCR) [27,29,46,47,48,49,50]. When investigating the roles of NS2A, NS4B, and 3′NCR in YFV dissemination in *Ae. aegypti* using chimeras employing the Asibi strain, McElroy et al. [28] concluded that the ability to disseminate of YFV in mosquitoes is a multigenic property. It has been suggested that restriction of YFV-17D replication in the midgut of *Ae. aegypti* occurs at the epithelial cell level and at a stage prior to the production of viral RNA, and that the low genetic diversity of the attenuated vaccine virus YFV-17D [45] compared to wild-type YFV contributes to its low infection ability and its inability to disseminate and be transmitted by *Ae. aegypti* [29]. Taken together, these data indicate that several non-mutually exclusive mechanisms and factors may explain the limited infection and lack of dissemination and transmission of attenuated vaccine viruses (17D or 17DD) in mosquitoes.

## 5. Conclusions

The low viremia presented by YF 17DD-vaccinated New World NHPs like lion tamarins combined with a low infection capacity and null dissemination ability of this attenuated virus in sylvatic and domestic mosquitoes suggests no uncontrolled transmission risks for this vaccine virus in nature.

## Figures and Tables

**Table 1:** Lion tamarin species, viremia at the time of mosquito feeding and number of mosquitoes by species examined at two incubation times (14 and 21 d.p.i.) after feeding on viremic YF 17DD-vaccinated lion tamarins.

Lion Tamarin Species (Individual Identification Number)	Viremia (FFU/mL)	Mosquitoes	Total	14 d.p.i.	21 d.p.i.	IR	DR
*Leontopithecus rosalia* (3617)	6.61 × 10^3^	*Sa. identicus*	15	-	15	0	0
		*Ae. aegypti*	34	34	-	0	0
*Leontopithecus rosalia* (2435)	5.37 × 10^3^	*Sa. albiprivus*	21	21	-	0	0
		*Ae. aegypti*	28	-	28	0	0
*Leontopithecus chrysomelas* (3504)	3.63 × 10^3^	*Sa. albiprivus*	13	13	-	0	NA
		*Ae. aegypti*	36	-	36	0	NA
*Leontopithecus* *chrysomelas* (3408)	3.54 × 10^3^	*Sa. albiprivus*	12	12	-	0	NA
		*Sa. identicus*	7	7	-	0	NA
		*Hg. leucocelaenus*	46	46	-	0	NA
		*Ae. aegypti*	17	17	-	0	NA
*Leontopithecus* *chrysomelas* (3644)	3.31 × 10^3^	*Sa. albiprivus*	30	30	-	0	NA
		*Sa. identicus*	15	15	-	0	NA
		*Hg. leucocelaenus*	53	53	-	0	NA
		*Ae. aegypti*	32	32	-	0	NA
*Leontopithecus* *chrysomelas* (3595)	2.75 × 10^3^	*Sa. albiprivus*	40	40	-	0	NA
		*Sa. identicus*	29	29	-	0	NA
		*Hg. janthinomys/capricornii*	5	5	-	0	NA
		*Hg. leucocelaenus*	42	42	-	0	NA
		*Ae. aegypti*	42	42	-	0	NA
*Leontopithecus rosalia* (3503)	1.95 × 10^3^	*Ae. aegypti*	30	30	-	0	NA
*Leontopithecus* *chrysopygus* (3533)	1.12 × 10^3^	*Sa. albiprivus*	11	11	-	0	NA
		*Ae. aegypti*	43	-	43	0	NA
*Leontopithecus* *chrysopygus* (2266)	1.05 × 10^3^	*Sa. identicus*	11	11	-	0	NA
		*Ae. aegypti*	38	38	-	0	NA
*Leontopithecus* *chrysomelas* (3110)	4.57 × 10^2^	*Ae. aegypti*	19	19	-	0	NA
*Leontopithecus* *chrysomelas* (3654)	1.91 × 10^2^	*Sa. albiprivus*	20	20	-	0	NA

Infection Rate (IR) = percentage of mosquitoes with infected bodies among the engorged individuals; Dissemination Rate (DR) = percentage of positive heads among individuals with positive bodies. NA = Not Analyzed.

**Table 2:** Parameters that determine vector competence evaluated in females of *Aedes aegypti* and *Hg. leucocelaenus* experimentally challenged orally with three different dilutions of the YF 17DD attenuated vaccine, after 14 days of incubation.

YF 17DD FFU/mL	Mosquito Species	Number of Tested Mosquitoes	IR N (%)	DR N (%)
1 × 10^4^	*Ae. aegypti* *Hg. leucocelaenus*	59 37	0 (0,0) 1 (2.7)	0 (0.0) 0 (0.0)
1 × 10^3^	*Ae. aegypti*	30	0 (0.0)	0 (0.0)
1 × 10^2^	*Ae. aegypti*	30	0 (0.0)	0 (0.0)
Total		156		

Infection Rate (IR) = percentage of mosquitoes with infected bodies among the engorged individuals; Dissemination Rate (DR) = percentage of positive heads among individuals with positive bodies.
